# Temporal Association in Hospitalizations for Tuberculosis, Invasive Pneumococcal Disease and Influenza Virus Illness in South African Children

**DOI:** 10.1371/journal.pone.0091464

**Published:** 2014-03-11

**Authors:** Ziyaad Dangor, Alane Izu, David P. Moore, Marta C. Nunes, Fatima Solomon, Natalie Beylis, Anne von Gottberg, Johanna M. McAnerney, Shabir A. Madhi

**Affiliations:** 1 Medical Research Council, Respiratory and Meningeal Pathogens Research Unit, Faculty of Health Sciences, University of the Witwatersrand, Johannesburg, South Africa; 2 Department of Paediatrics, Faculty of Health Sciences, University of the Witwatersrand, Johannesburg, South Africa; 3 Department of Science and Technology/National Research Foundation: Vaccine Preventable Diseases, University of the Witwatersrand, Johannesburg, South Africa; 4 Mycobacteriology Referral Laboratory, National Health Laboratory Service, Johannesburg, South Africa; 5 Center for Respiratory and Meningitis Diseases, National Institute for Communicable Diseases: A Division of National Health Laboratory Service, Sandringham, South Africa; National Institutes of Health, United States of America

## Abstract

**Introduction:**

The seasonal variability in hospitalization for tuberculosis may in part relate to super-imposed bacterial or predisposing respiratory viral infections. We aimed to study the temporal association between hospitalization for culture-confirmed pulmonary tuberculosis (PTB), invasive pneumococcal disease (IPD) and influenza virus epidemics in South African children.

**Methods:**

We undertook a retrospective analysis which examined seasonal trends, from 2005 to 2008, for hospitalization for culture-confirmed PTB and IPD among children in relation to the influenza epidemics in Soweto, South Africa. Original time-series of the influenza virus epidemics and hospitalization rates for PTB and IPD were decomposed into three components: a trend cycle component, a seasonal component and an irregular component using the X-11 seasonal adjustment method. To compare the seasonality amongst the three series, the trend and irregular components were removed and only seasonal components examined.

**Results:**

Across the study period, the influenza virus epidemics peaked during May to July (winter) months, which was closely followed by an increase in the incidence of hospitalization for IPD (August to October) and PTB (August to November).

**Discussion:**

Within- and between-year temporal changes associated with childhood TB hospitalization may in part be driven by factors which influence temporal changes in pneumococcal disease, including potential variability in the severity of influenza virus epidemics in temperate climates. The dynamics of the interplay between the host and these infectious agents appears to be complex and multifactorial.

## Introduction

We have previously reported seasonal variability in hospitalization for culture-confirmed tuberculosis (TB) in South African children [1]. This temporal variability in incidence of culture-confirmed TB between and within study years, may in part relate to hospitalization for TB being precipitated by concurrent co-infections, including due to pneumococcus or due to increased immunological susceptibility following influenza virus [2]–[5]. Consequently, the within- and between-year temporal changes associated with childhood TB hospitalization may in part be driven by factors which influence temporal changes in pneumococcal disease, including potential variability in the severity of influenza virus epidemics in temperate climates.

A number of experimental models have studied the interaction between TB and invasive pneumococcal disease (IPD) [6]–[8], as well as influenza and IPD [9]–[11]. The interactions between these pathogens may relate to changes in the host epithelial barriers and failure of the innate or adaptive immune systems through dysregulation of chemical modulators, resulting in a window period of vulnerability in the exposed host [12]. This interaction has also been examined epidemiologically, [2], [3], [13], [14] including in the context of mortality observed during the influenza pandemics [15]–[17]. To date, no population-based study has interrogated the possible interaction between these three pathogens in children.

The aim of this study was to evaluate whether there is seasonal pattern between influenza epidemics and hospitalization for IPD and TB in South African children.

## Methods

### Study setting

We undertook a retrospective study, to compare the incidence of culture-confirmed pulmonary TB (PTB) and culture-confirmed IPD among children hospitalized from 2005 to 2008 in Soweto, Gauteng, South Africa in relation to background surveillance data on influenza epidemics in the study-setting. The population of Soweto in 2005 included approximately 1.12 million mainly black urban South Africans, including 120,000 children under five years of age [18]. An estimated 90% of individuals in Soweto requiring hospitalization are admitted to Chris Hani-Baragwanath Academic Hospital (CHBAH), the only public-hospital in the study setting. All public-based health care, including hospitalization of children, is provided free-of-charge by the State.

### PTB in study population

The standard-of-care of investigating for TB in children hospitalized for pneumonia or with other clinical signs and symptoms of TB, at CHBAH included a low threshold for performing 2–3 gastric washings or induced-sputum sample collections for *Mycobacterium tuberculosis* (MTB) culture. Identification of MTB was undertaken by the National Health Laboratory Service (NHLS), which used the WHO recommended method of specimen processing using N-acetyl-L-cysteine-NaOH, for culture of MTB from sputum. These samples were incubated in the MGIT (Mycobacterium Growth Indicator Tube) 960™ TB System (Becton Dickinson, Sparks, Maryland). Details of the study procedures have been described [1].

### Invasive pneumococcal disease in the study population

An IPD episode was defined as identification of pneumococcus from a normally sterile site (e.g., blood, cerebrospinal fluid [CSF], pleural fluid or joint fluid). The standard culture methods at the hospital included blood cultured for pneumococcal growth with the BacT/Alert microbial detection system (Organon Teknika, Durham, NC). Blood culture specimens which flagged positive but were bacterial culture negative, and that were macroscopically chocolate-coloured with or without pleomorphic Gram-positive cocci on microscopy, were tested using a latex agglutination kit (Wellcogen Bacterial Antigen Kit, Remel Europe Ltd, Dartford, UK). CSF, peritoneal, pericardial and other fluid samples were processed according to standard procedures. Cases of IPD at CHBAH were identified through the GERMS-SA (Group for Enteric, Respiratory and Meningeal Disease Surveillance) database. During the study period, pneumococcal vaccines were unavailable as part of the national immunization program.

### Influenza epidemics during the study period

The influenza epidemic in South Africa generally prevails in the winter (May to September). Influenza surveillance data is collated by the Viral Watch Program (VWP), a division of the National Institute for Communicable Diseases [19]. The VWP monitors influenza patterns in more than 170 sites country-wide, predominantly (89–92%) in primary health care settings, and the methods used to test for influenza virus have been described [19]. Between 2005 and 2008, 5424 samples were received (Ages: 1 month to 84 years), 29% in children under 15 years of age [19]. The dominant influenza strains in the four years were: 2005, A(H1N1) 317/564 (56%) detections; 2006, A(H3N2) 389/491 (79%) detections; 2007, A(H3N2) 240/556 (43%) (the remainder split more or less evenly between A(H1N1) and B); 2008, A(H1N1) 310/390 (80%) detections [20].

### HIV in study population

The prevalence of HIV infection in Gauteng province was estimated to be 2.46% in children <15 years of age between 2005 and 2008 [21]. The standard-of-care for confirmation of HIV infection status in children is based on an enzyme-linked immunosorbent assay (ELISA) test in children older than 18 months of age and on an HIV-1 polymerase chain reaction (PCR) in children <18 months of age with a history of maternal HIV-exposure or a reactive HIV ELISA test. HIV-infected children in Soweto during the course of the study period primarily received HIV/AIDS care at one of two pediatric HIV clinics based at CHBAH. Criteria for admission to the hospital and investigation for TB of HIV-infected and -uninfected children was at the discretion of attending physicians.

### Statistical analysis

Trends in TB, IPD and influenza incidence rates were examined using Proc X11 in SAS v9.2 (SAS Institute Inc., Cary, NC). Original time series of influenza virus circulation (all ages), hospitalizations of children for PTB and IPD incidence rates were decomposed into three components: a trend cycle component, a seasonal component and an irregular component using the X-11 seasonal adjustment method [22].

To compare the seasonality amongst the three series, the trend and irregular components were removed and only the seasonal components were examined. Figures displaying the decomposition of the three series are included as figures S1, S2, S3. These seasonal components are quantities added to the trend and irregular components to obtain the original series. The main interest was not in the amplitude of the seasonal component, but in the timing of the peaks from year to year and how this timing compares amongst the three diseases.

The study was approved by the Medical Advisory Committee at CHBAH and by the by the Human Research Ethics Committee (HREC), University of Witwatersrand. Parental consenting was waivered by the HREC for this retrospective study.

## Results

Six-hundred and sixty-seven children <15 years old were hospitalized with culture-confirmed PTB from 2005 to 2008, among whom the prevalence of HIV remained consistent (53–59%), although the frequency of HIV testing increased over time. Most children were underweight for age with a median weight-for-age Z-score of −2.80 (Range: −8.89–1.87). The highest burden of PTB was in children <2 years of age, with an overall median age of 1.72 years (Range: 0.25–14.25 years), Table 1. During the same period, 636 children were admitted for IPD, of whom 367 (57.7%) were HIV-infected. The majority (53.6%) of IPD cases also occurred in children <2 years of age, at a median age of 1.70 years (Range 0.25–14.68 years), Table 1. Twelve children, nine of whom were HIV-infected, were hospitalized for IPD and PTB within two weeks of either diagnosis. Six of these children were in the 3 month to <2 year age group, three were 2 to <5 years of age and three were 5 to <15 years of age.

**Table 1:** Demographic information on children hospitalized with culture-confirmed pulmonary tuberculosis (PTB) and invasive pneumococcal disease (IPD).

Year	2005	2006	2007	2008
**Total cases of culture-confirmed PTB**	**216**	**211**	**136**	**104**
** HIV-Infected**	127 (58.8%)	112 (53.1%)	80 (58.8%)	55 (52.9%)
** HIV-uninfected**	56 (25.9%)	68 (32.2%)	41 (30.2%)	42 (40.4%)
** HIV-unknown**	33 (15.3%)	31 (14.7%)	15 (11.0%)	7 (6.7%)
**Age (%)**				
** 3 m-<2 years**	123 (56.9%)	120 (56.9%)	70 (51.5%)	47 (45.2%)
** 2-<5 years**	29 (13.4%)	38 (18.0%)	23 (16.9%)	26 (25.0%)
** 5-<15 years**	64 (29.7%)	53 (25.1%)	43 (31.6%)	31 (29.8%)
** Median (Range)**	1.47 (0.25–13.93)	1.36 (0.25–14.25)	1.87 (0.25–13.94)	2.19 (0.25–13.70)
**Gender (%)**				
** Male**	112 (51.9%)	114 (54.0%)	76 (55.9%)	52 (50.0%)
** Female**	104 (48.1%)	97 (46.0%)	60 (44.1%)	52 (50.0%)
**Weight-for-age Z-scores**				
** Median (Range)**	−2.81 (−7.14–1.87)	−2.74 (−7.83–4.39)	−3.18 (−8.89–1.8)	−2.89 (−7.31–1.44)
**Total cases of culture-confirmed IPD**	**188**	**155**	**156**	**137**
** HIV-Infected**	109 (58.0%)	100 (64.5%)	87 (55.8%)	71 (51.8%)
** HIV-uninfected**	37 (19.7%)	31 (20.0%)	49 (31.4%)	59 (43.1%)
** HIV-unknown**	42 (22.3%)	24 (15.5%)	20 (12.8%)	7 (5.1%)
**Age (%)**				
** 3 m-<2 years**	98 (52.1%)	78 (50.3%)	81 (51.9%)	84 (61.3%)
** 2-<5 years**	44 (23.4%)	35 (22.6%)	34 (21.8%)	18 (13.1%)
** 5-<15 years**	46 (24.5%)	42 (27.1%)	41 (26.3%)	35 (25.6%)
** Median(Range)**	1.82 (0.27–14.68)	1.97 (0.29–13.55)	1.79 (0.25–13.7)	1.09 (0.27–14.32)
**Gender (%)**				
** Male**	90 (47.9%)	73 (47.1%)	81 (51.9%)	77 (56.2%)
** Female**	92 (48.9%)	81 (52.3%)	73 (46.8%)	60 (43.8%)
** unknown**	6 (3.2%)	1 (0.6%)	2 (1.3%)	0 (0%)

### Seasonality

**Figure 1:**
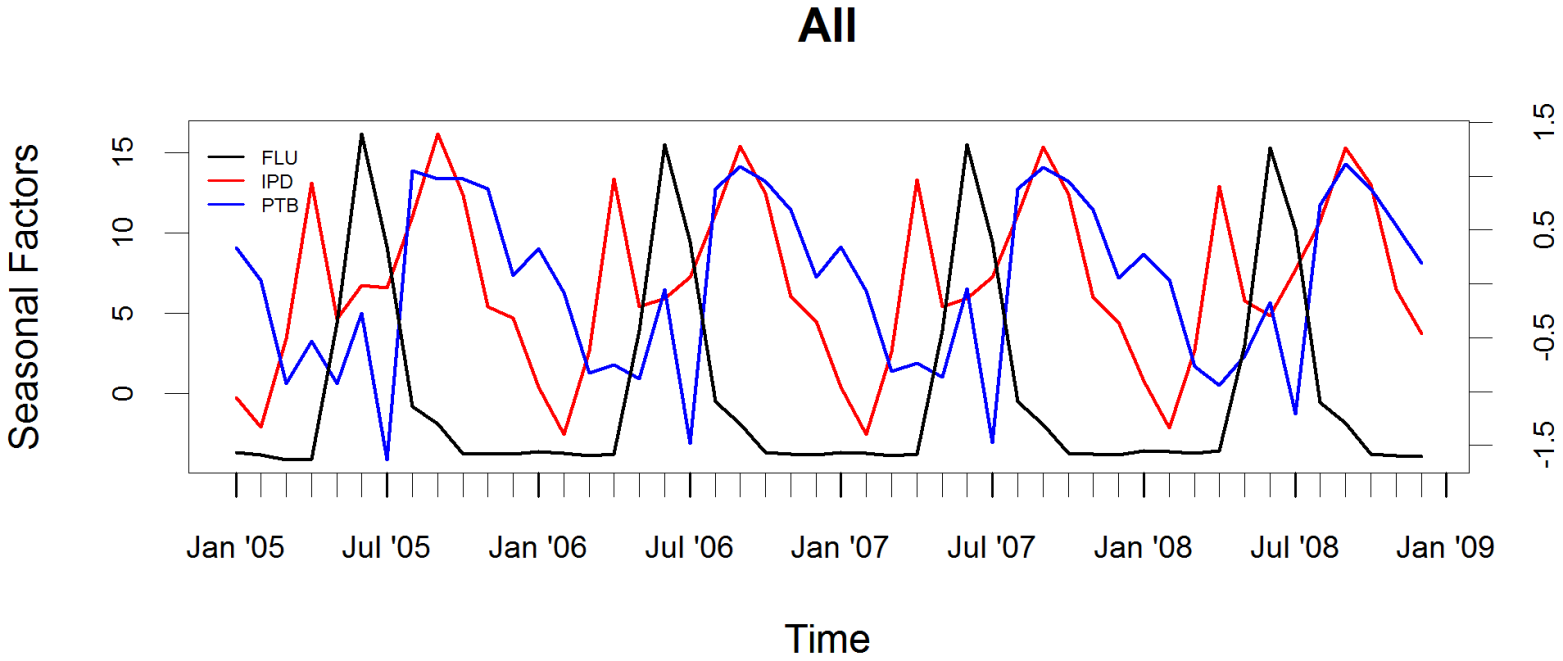
Seasonality of FLU, IPD and PTB in All children. Seasonal factors of influenza (FLU) virus overall circulation and association to hospitalization for invasive pneumococcal disease (IPD) and culture-confirmed pulmonary tuberculosis (PTB) in children. The axis for seasonal factors for influenza is on the left and for IPD and PTB is on the right.

The seasonality of influenza virus illness and hospitalization for IPD and PTB are depicted in Figure 1. Although the X-11 method allows for evolving seasonal components, the seasonality factors were similar from year to year. The pattern of influenza virus infections peaked mainly in May to September of each year. Influenza peaks were followed by increases in the number of children hospitalized with IPD and subsequently PTB. There was a time lag from the influenza virus peaks to the peak in hospitalization for IPD (3 months) and PTB (2–3 months). Influenza activity peaked in June, IPD in September, whilst PTB peaked during August (in 2005) and September in 2006–2008. Notably, a lesser peak in IPD occurred in April which subsequently waned over the influenza period before peaking again three months after the peak of the influenza season. The peaks in hospitalization of IPD and PTB tended to occur at similar time points.

**Figure 2:**
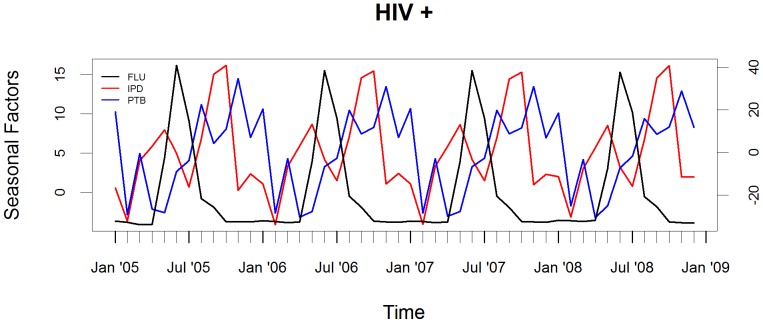
Seasonality of FLU, IPD and PTB in HIV-infected children. Seasonal factors of influenza (FLU) virus overall circulation and association to hospitalization for invasive pneumococcal disease (IPD) and culture-confirmed pulmonary tuberculosis (PTB) in HIV-infected children. The axis for seasonal factors for influenza is on the left and for IPD and PTB is on the right.

**Figure 3:**
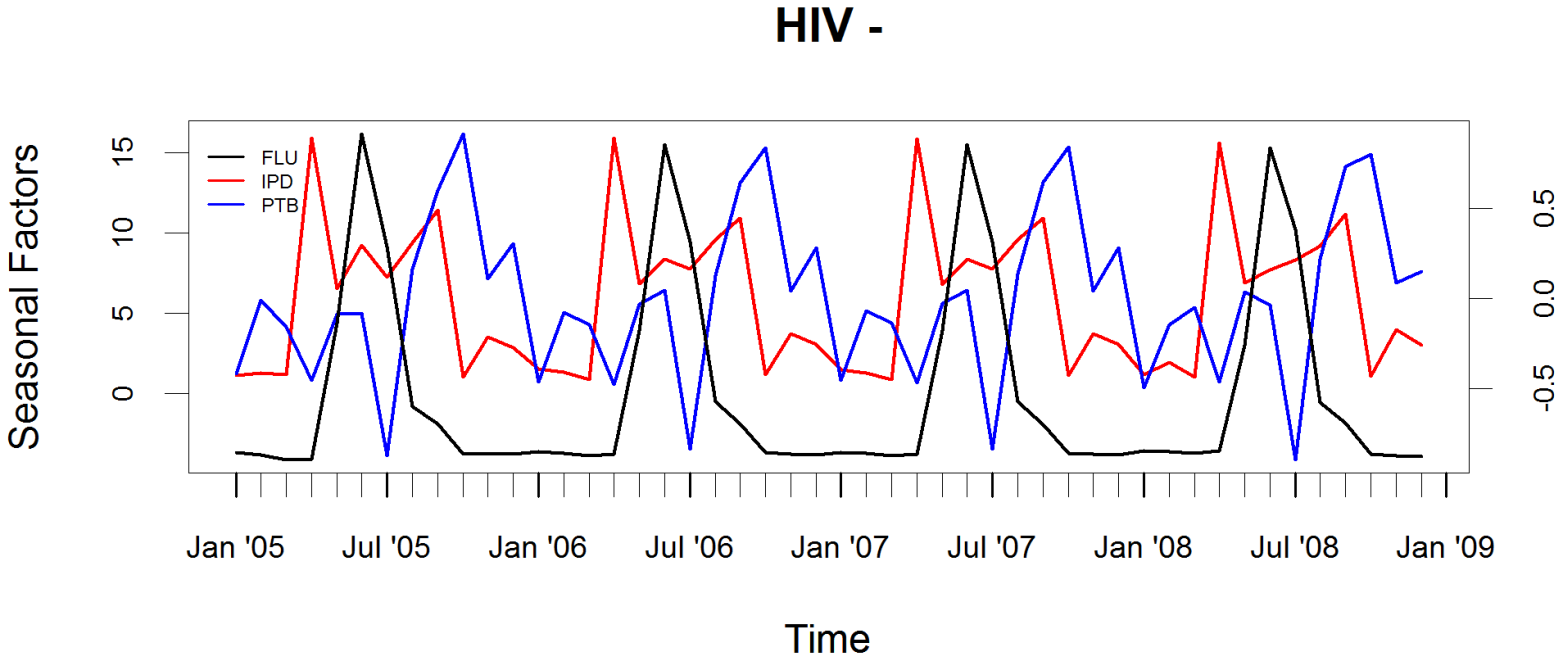
Seasonality of FLU, IPD and PTB in HIV-uninfected children. Seasonal factors of influenza (FLU) virus overall circulation and association to hospitalization for invasive pneumococcal disease (IPD) and culture-confirmed pulmonary tuberculosis (PTB) in HIV-uninfected children. The axis for seasonal factors for influenza is on the left and for IPD and PTB is on the right.

The peaks in IPD and PTB relative to the influenza peak occurred in October (4 months later) and November (5 months later), respectively, in HIV-infected children, Figure 2. In contrast, the peak in hospitalization for IPD in HIV-uninfected children occurred prior to the peak in influenza, Figure 3.

Furthermore, we analysed the lagged month-by-month correlations between the raw data on influenza, IPD and PTB rates. We found the highest correlations between influenza and IPD or PTB, when the rate of influenza in June coincided with the rate of IPD in August and with PTB in September/October. The correlation between IPD and PTB was highest when PTB was lagged by 1 month.

## Discussion

We observed a seasonal pattern in a time-sequenced manner of influenza virus activity, followed by a peak in IPD and PTB. This sequential risk of pneumococcal disease following influenza illness was first hypothesized in reference to the 1918/19 pandemic, in which deaths during the influenza pandemic occurred 7–10 days following the influenza illness and was attributed to secondary bacterial infection [23]. The vulnerability of the host to pneumococcal disease following influenza virus during this “window period” is likely to be multifactorial, and include host local and systemic immune factors [12]. In our study, however, we report a peak in IPD three months following the peak in influenza virus activity, making it less likely that the susceptibility to IPD was mainly precipitated by influenza virus infection. In contrast, the peak observed in hospitalization for culture-confirmed PTB, which followed 2–3 months following the peak in influenza virus circulation, could be explained in children less than 5 years of age being due to primary infection by *Mycobacterium tuberculosis* (MTB), following which it takes 6 weeks to three months for primary PTB to manifest [24]. This was corroborated partly in animal-model studies in which mice inoculated with influenza virus and then with MTB three weeks following influenza exposure, were at increased risk of developing TB [5]. We speculate that children living in close proximity to adults with infectious PTB may be at enhanced risk of primary infection with MTB when the adult source case is co-infected with a respiratory virus (e.g., influenza) and so more likely to be aerosolizing tubercle bacilli.

In older individuals, PTB may be due to reactivation of underlying latent TB infection because of immunosuppression induced by influenza virus infection, [25] with the influenza-virus induced immune suppression increasing the susceptibility of individuals with underlying TB to developing acute pneumonia [2].

The prolonged time-lag in peak of influenza virus activity and subsequent IPD in our study is not consistent with observations of others (<1 month), or that indicated by animal-model-studies [2], [15], [23], [26], [27]. It is, however, possible that influenza virus epidemics increased susceptibility to nasopharyngeal acquisition of new pneumococcal serotypes, for which the risk of disease is greatest up to two months post-acquisition [28]. Alternately, other factors may contribute to this temporal association between the peak in influenza virus activity and IPD in settings such as South Africa. In particular, the possible increased susceptibility to developing TB following influenza epidemics, may lend itself to underlying immune suppression which subsequently increases the susceptibility to developing IPD in the South African context, especially with its high burden of HIV and TB co-infection. Previous studies conducted in high HIV prevalent settings have reported an increased burden of IPD and PTB disease in HIV-infected children [1], [29]. Furthermore, almost two thirds of hospitalized PTB and IPD cases with a known HIV status were HIV-infected in our study period.

To our knowledge, this is the first study to describe the seasonality among all three of these pathogens, the plausibility of which is corroborated by experimental models, vaccine probe studies and epidemiological observations during influenza pandemics. The possible mechanisms supporting an increased host susceptibility to IPD following influenza virus infection are evident from experimental animal-model studies, which have been reviewed in detail [9]–[11]. The dynamics of the interplay between the host and the infectious agent is complex and multifactorial and include disruptions in the physical barriers and an imbalance in the pro-inflammatory (interleukin [IL]-1, IL6, tumor necrosis factor [TNF] and gamma-interferon [INFy]) and anti-inflammatory (IL10, IL17) cytokine/chemokine pathways which disrupt the recruitment of cellular elements of the innate immune response, including macrophages and neutrophils. Although this process may be beneficial to the host in establishing long-term immunity at the time of the pneumococcal infection, it may increase susceptibility to TB.

Specifically, idiosyncrasies in the regulation of Toll-like receptors (TLRs) and INFγ are common to influenza illness, pneumococcal disease and TB. TLRs are pattern recognition receptors expressed by macrophages, dendritic cells and lymphocytes. Certain TLRs are down-regulated by influenza virus, thus resulting in a failure of macrophages to activate NF-kB and chemokines which in turn result in less neutrophil recruitment [9], [10]. TLRs, particularly TLR-2, are also important in the host response to TB [6]. Studies have demonstrated that an increased predisposition to TB can occur by altering the encoding proteins that signal TLR activity [7]. Neutrophils have also been shown to protect mice against TB [8], so it is plausible that perturbations in TLR functioning with its effect on neutrophil recruitment, may play a role in predisposition to MTB disease. On the other hand, INFγ produced by lymphocytes is an important component of the host defense to viruses as well as intracellular pathogens such as MTB. Bronchiolar lavage on mice with influenza and subsequently infected with pneumococcus had increased levels of INFγ as opposed to mice with human metapneumovirus (hMPV) and subsequently infected with pneumococcus [26]. INFγ produced in response to viral infections results in inadequate bacterial clearance from the lung by macrophages in-vivo and in-vitro [30]. Furthermore, high concentrations of INFγ disrupted macrophage clearance in the alveolus [10].

Our clinical understanding of the association between influenza epidemics and susceptibility to bacterial infections at a population level arise from ecological studies. It is reported that most deaths during the influenza pandemic of 1918/19 were related to secondary bacterial infection, including pneumococcus [23], [31] with a median time of death of 7–10 days [23]. In 14 studies addressing fatal bacterial pneumonia in the 1918/1919 pandemic, pneumococci was identified in 81% of 249 positive cultures [15]. Furthermore, pneumococcus was identified in 24% of postmortem lung biopsies [31]. During the 2009 H1N1 influenza pandemic, 36 fatalities were reported in children in the United States, 10 (43%) of whom had bacterial co-infection, three with pneumococcus [16]. In countries where pneumococcal conjugate vaccine (PCV) was not routinely given to children, pneumococcal bacteremia was highest in children <4 years of age [32]. An increase in the number of TB-related deaths was also noted during the 1918/19 pandemic [4], [33]. In South Africa, where the burden of TB continues to remain high, 10% of fatalities associated with the 2009 H1N1 influenza virus pandemic had concurrent TB [17].

Furthermore, PCV probe studies also corroborated the suggestion of an interaction between pneumococcal disease and both influenza illness [14], and TB [2]. Notably, PCV-vaccinated children were less likely to be hospitalized for pneumonia attributable to respiratory viruses, including influenza virus (45% efficacy) [14], [34], and had a reduced likelihood of being hospitalized for culture-confirmed PTB (43% efficacy) compared to placebo recipients [2]. These clinical observations were attributed to hospitalizations for influenza-associated pneumonia and culture-confirmed PTB possibly having been precipitated by co-infection with pneumococcus [2]. The interaction between IPD and TB is further supported by case reports in HIV-infected adults [35], [36].

A limitation of our study is that it focused solely on hospitalized cases. Our study could not measure whether there may have been any changes in clinician behavior in terms of referral, admission and investigating children with TB over time. The influenza data is based on community surveillance; however, although not specific to the Soweto population, the seasonality of influenza is unlikely to be different in this region as most of the samples were collected from the broader Johannesburg region [19]. Although we could not establish the occurrence of all three disease processes in the same individual, at least twelve children had IPD and culture-confirmed PTB within two weeks of either diagnosis during their hospitalization. We observed a peak in IPD prior to the influenza season which may be explained by other respiratory viruses precipitating an increase in IPD hospitalization, particularly RSV from early March in this setting [13], [37], [38].

In conclusion, our study indicates a temporal association between influenza illness, pneumococcal disease and TB. Coincidently, these peaks in hospitalization followed the influenza seasonal peaks, more so in HIV-infected children. The peaks in hospitalization for IPD and PTB were largely synchronous over the study period. It is conceivable that interventions against any one of these, e.g. vaccination against influenza virus or pneumococcal disease, may alter the epidemiology of hospitalization to another in our setting and settings such as ours (low-income, high-density with a high background HIV and TB prevalence). This has already been partly corroborated by our observations on PCV and its effect on hospitalization rates for influenza-associated pneumonia and culture-confirmed PTB [2], [14].

## Supporting Information

Figure S1 **Decomposition of the time series for influenza (FLU) virus data into the original (raw data), seasonal, trend and irregular components.**
(TIFF)

Figure S2 **Decomposition of the time series for invasive pneumococcal disease (IPD) data into the original (raw data), seasonal, trend and irregular components.**
(TIFF)

Figure S3 **Decomposition of the time series for culture-confirmed pulmonary tuberculosis (PTB) data into the original (raw data), seasonal, trend and irregular components.**
(TIFF)
